# Cadmium Exposure and Cancer Mortality in a Prospective Cohort: The Strong Heart Study

**DOI:** 10.1289/ehp.1306587

**Published:** 2014-02-14

**Authors:** Esther García-Esquinas, Marina Pollan, Maria Tellez-Plaza, Kevin A. Francesconi, Walter Goessler, Eliseo Guallar, Jason G. Umans, Jeunliang Yeh, Lyle G. Best, Ana Navas-Acien

**Affiliations:** 1 Department of Environmental Health Science, and; 2 Welch Center for Prevention, Epidemiology and Clinical Research, Johns Hopkins Bloomberg School of Public Health, Baltimore, Maryland, USA; 3 Environmental Epidemiology and Cancer Unit, National Center for Epidemiology, Carlos III Institute of Health, Madrid, Spain; 4 Consortium for Biomedical Research in Epidemiology & Public Health (CIBER en Epidemiología y Salud Pública–CIBERESP), Madrid, Spain; 5 Fundacion de Investigacion del Hospital Clinico de Valencia-INCLIVA, Valencia, Spain; 6 Institute of Chemistry, Analytical Chemistry, Karl-Franzens University, Graz, Austria; 7 Department of Epidemiology, Johns Hopkins Bloomberg School of Public Health, Baltimore, Maryland, USA; 8 Department of Medicine, Johns Hopkins Medical Institutions, Baltimore, Maryland, USA; 9 MedStar Health Research Institute, Hyattsville, Maryland, USA; 10 Georgetown-Howard Universities Center for Clinical and Translational Science, Washington, DC, USA; 11 Center for American Indian Health Research, College of Public Health, University of Oklahoma Health Sciences Center, Oklahoma City, Oklahoma, USA; 12 Missouri Breaks Industries Research Inc., Timber Lake, South Dakota, USA; 13 Department of Oncology, Johns Hopkins Medical Institutions, Baltimore, Maryland, USA

## Abstract

Background: Cadmium (Cd) is a toxic metal classified as a human carcinogen by the International Agency for Research on Cancer.

Objective: We evaluated the association of long-term Cd exposure, as measured in urine, with cancer mortality in American Indians from Arizona, Oklahoma, and North and South Dakota who participated in the Strong Heart Study during 1989–1991.

Methods: The Strong Heart Study was a prospective cohort study of 3,792 men and women 45–74 years of age who were followed for up to 20 years. Baseline urinary Cd (U-Cd) was measured using inductively coupled plasma mass spectrometry. We assessed cancer events by annual mortality surveillance.

Results: The median (interquintile range) U-Cd concentration was 0.93 (0.55, 1.63) μg/g creatinine. After adjusting for sex, age, smoking status, cigarette pack-years, and body mass index, the adjusted hazard ratios (HRs) comparing the 80th versus the 20th percentiles of U-Cd were 1.30 (95% CI: 1.09, 1.55) for total cancer, 2.27 (95% CI: 1.58, 3.27) for lung cancer, and 2.40 (95% CI: 1.39, 4.17) for pancreatic cancer mortality. For all smoking-related cancers combined, the corresponding HR was 1.56 (95% CI: 1.24, 1.96). Cd was not significantly associated with liver, esophagus and stomach, colon and rectum, breast, prostate, kidney, or lymphatic and hematopoietic cancer mortality. On the basis of mediation analysis, we estimated that the percentage of lung cancer deaths due to tobacco smoking that could be attributed to Cd exposure was 9.0% (95% CI: 2.8, 21.8).

Conclusions: Low-to-moderate Cd exposure was prospectively associated with total cancer mortality and with mortality from cancers of the lung and pancreas. The implementation of population-based preventive measures to decrease Cd exposure could contribute to reducing the burden of cancer.

Citation: García-Esquinas E, Pollan M, Tellez-Plaza M, Francesconi KA, Goessler W, Guallar E, Umans JG, Yeh J, Best LG, Navas-Acien A. 2014. Cadmium exposure and cancer mortality in a prospective cohort: the Strong Heart Study. Environ Health Perspect 122:363–370; http://dx.doi.org/10.1289/ehp.1306587

## Introduction

Cadmium (Cd) is a widespread metal that is highly toxic to humans. Cd pollution in soil, air, and water is ubiquitous because of Cd use in industrial products (e.g., batteries, coatings, plastic stabilizers), contamination of phosphate fertilizers, and release from motor vehicle fuel combustion and tire wear (Agency for Toxic Substances and Disease Registry 2011). Soil contamination is a major health problem because grains and leafy and root vegetables bioconcentrate Cd, resulting in major sources of Cd exposure through diet and smoking.

Cd is classified as a human carcinogen by the International Agency for Research on Cancer (IARC 1993). Cd exposure has been associated with lung cancer incidence in a population living in a Cd-polluted area (Nawrot et al. 2006) and with lung cancer incidence and mortality in occupationally exposed populations (Jarup et al. 1998; Park et al. 2012). In experimental models, Cd acts as an endocrine disruptor (Martin et al. 2002; Siewit et al. 2010), supporting the hypothesis that this metal can potentially induce the development of hormone-dependent tumors in humans, including those of the breast, uterus, and prostate (Akesson et al. 2008; Benbrahim-Tallaa et al. 2009; Bertin and Averbeck 2006). In occupationally exposed women, Cd has been associated with increased breast cancer incidence (Pollán and Gustavsson 1999) and breast cancer mortality (Cantor et al. 1995). In other studies, however, occupational Cd exposure was not associated with breast cancer incidence or mortality (Jarup et al. 1998; Kauppinen et al. 2003). Some evidence also suggests that occupational Cd exposure may be a risk factor for kidney (Il’yasova and Schwartz 2005) and pancreatic cancers (Schwartz and Reis 2000).

Less is known about the carcinogenicity of Cd at low-to-moderate levels of exposure. In the Third National Health and Nutrition Examination Survey (NHANES) (1988–1994), urinary Cd (U-Cd) was associated with total cancer mortality over a period of 13.5 years of follow-up (Adams et al. 2012). In men, Cd was associated with cancers of the lung and pancreas and with non-Hodgkin lymphoma, but not with prostate cancer; whereas in women, Cd was associated with cancers of the lung, ovaries, and uterus and with leukemia, but not with breast cancer (Adams et al. 2012). Cd exposure, however, has been associated with breast cancer in women from general populations in Sweden (Julin et al. 2012a) and the United States (Gallagher et al. 2010; McElroy et al. 2006) and with endometrial cancer (Akesson et al. 2008).

Cancer is the second leading cause of death in American Indians (Centers for Disease Control and Prevention 2013). During 1999–2008, cancer death rates declined by > 1% per year in every American ethnic/racial group with the exception of American Indians (Siegel et al. 2012). Few studies, however, have evaluated the cancer burden and its determinants in this population. The main objective of the present study was to evaluate the association of U-Cd concentrations with overall and site-specific cancer mortality in American Indian adults who participated in the Strong Heart Study (SHS) during 1989–1991 and were followed through 2008. In the present study, we assume that U-Cd is a biomarker of long-term Cd exposure (Jarup and Akesson 2009). In addition to diet and smoking, other sources of Cd exposure for American Indian populations include living in the vicinity of industrial sites and mining areas (Moon et al. 1986; Schmitt et al. 2006), surface dust in jewelry-making homes (Gonzales et al. 2004), and small-scale motor vehicle repair (Yassin and Martonik 2004).

## Methods

*Study population*. From 1989 through 1991, men and women 45–75 years of age from 13 American Indian communities were invited to participate in the SHS. In Arizona and Oklahoma, every eligible person was invited; whereas in North and South Dakota, a cluster sampling technique was used (Lee et al. 1990). Among those invited, 62% agreed to participate and were evaluated at baseline (Stoddart et al. 2000), with a final sample of 4,545 participants. We excluded 580 participants due to insufficient urine available for metal analysis, 151 participants without information on smoking, 15 participants without body mass index (BMI) determinations, and 7 participants with missing information on alcohol consumption or education level, leaving 3,792 participants for these analyses. The SHS protocol was approved by the Institutional Indian Health Service Review Boards and by the participating Indian Communities. All participants provided oral and written informed consent.

*Baseline data collection*. Study visits were performed by trained and certified examiners following a standard protocol (Lee et al. 1990) and included a questionnaire (sociodemographic factors, smoking status, and medical history), a physical examination (height, weight, and blood pressure), and blood and urine collection. Participants having smoked ≥ 100 cigarettes in their lifetime and still smoking at baseline were considered current smokers. Past smoking was defined as noncurrent smokers who had smoked > 100 cigarettes in their lifetime. Cigarette pack-years were calculated as the number of cigarette packs smoked per day times the number of years the person smoked. Current alcohol consumption was defined as any alcohol use within the past year. Former alcohol consumption was defined as no use of any alcohol during the last year but previous use of > 12 drinks of alcohol. Menopause was defined as the absence of a menstrual cycle for ≥ 12 months, a history of hysterectomy and oophorectomy, or a history of hysterectomy without oophorectomy and an age of ≥ 53 years. Hypertension was defined as mean systolic blood pressure ≥ 140 mmHg, mean diastolic blood pressure ≥ 90 mmHg, or use of an antihypertensive medication. Plasma creatinine was measured by an alkaline picrate rate method to estimate glomerular filtration rate (Levey et al. 2009), and urinary creatinine was measured by an automated alkaline picrate methodology (Lee et al. 1990).

*U-Cd determinations*. The analytical methods used to measure U-Cd have been described in detail by Scherer and Barkemeyer (1983). In summary, we measured Cd in spot urine samples using inductively coupled plasma mass spectrometry (Agilent 7700x ICP-MS; Agilent Technologies, Waldbronn, Germany). The limit of detection (LOD) for U-Cd was 0.015 μg/L and the interassay coefficient of variation was 8.7%. We imputed the U-Cd concentration for one sample that was below the LOD as the LOD divided by the square root of 2.

*Cancer mortality follow-up*. Death certificates were obtained from each state’s Department of Health. If the death certificate indicated that an autopsy had been performed, the medical examiner’s report was obtained (Lee et al. 2006). Primary and contributing causes of death were recorded according to the *International Classification of Diseases, 9th Revision* (ICD-9) (World Health Organization 1977). In addition to total cancer, we evaluated the following specific cancers: esophagus and stomach (ICD-9 codes 150–151), colon and rectum (codes 153–154), liver and intrahepatic bile ducts (code 157), gall bladder and extrahepatic bile ducts (code 156), bronchus and lung (codes 162.2–162.9) (hereafter referred to as lung cancer), breast (code 174), prostate (code 185), kidney (code 189.0), and lymphatic and hematopoietic tissue (codes 200–208). Finally, we evaluated cancers with sufficient evidence of a causal association with tobacco smoking according to the IARC (2012) as a single group, including cancers of the lip, oral cavity, and pharynx (codes 140–149), esophagus (code 150), stomach (code 151), colon and rectum (codes 153–154), liver (code 155), pancreas (code 157), larynx (code 161), trachea, bronchus, and lung (code 162), cervix (code 180), bladder (code 188), and kidney (code 189) and myeloid leukemia (code 205). The SHS uses tribal records, death certificates, and direct annual contact with participants and their families to assess health outcomes and vital status over time. Follow-up for mortality was complete for 99.8% of the study population. We calculated follow-up from the date of baseline examination to the date of death or 31 December 2008, whichever occurred first. The mean follow-up time among participants who did not develop cancer was 17.2 years.

*Statistical methods*. U-Cd concentrations were markedly right-skewed and natural log (ln)–transformed for statistical analyses. To account for urine dilution in spot urine samples, we divided Cd by urinary creatinine. We conducted statistical analyses using Stata (version 11.2; StataCorp, College Station, TX, USA).

We assessed the prospective association between creatinine-corrected Cd concentrations and cancer mortality (overall and site-specific) using Cox proportional hazards models with age as the time scale and individual follow-up starting times (age at baseline examination) treated as staggered entries. This approach effectively adjusts for age. We visually evaluated the proportional hazards assumption based on Schoenfeld residuals and did not observe any major departures from proportionality (data not shown). To account for region, the nonparametric underlying baseline hazards were allowed to differ by study region using the strata command. We estimated associations with Cd modeled as tertiles, with the lowest tertile as the reference level of exposure. For pancreatic cancer, there was only one case in the first Cd tertile; therefore, we combined the first and second tertiles. We also modeled ln-transformed Cd as a continuous variable and derived hazard ratios (HRs) comparing the 80th versus the 20th percentiles [i.e., the interquintile range (IQR)] of its distribution. In addition, in a third set of models, we estimated associations with Cd modeled as restricted cubic splines with knots at the 10th, 50th, and 90th percentiles.

All Cox proportional hazard models accounted for age and region (model 1). Model 2 was further adjusted for sex, smoking status, cigarette pack-years, and baseline BMI. Model 2 was further adjusted for baseline menopausal status (premenopausal, postmenopausal), hormone replacement therapy (current, past, never users), and parity (0, 1–2, 3–4, ≥ 5) when breast cancer was the outcome of interest (Jain 2013; Sasco 2001; Vahter et al. 2004), and for hypertension (no, yes) and glomerular filtration rate (continuous) when kidney cancer was the studied outcome (Brennan et al. 2008; Choi et al. 2005). To evaluate the consistency of our findings across subgroups, we performed separate exploratory models for total cancer mortality and smoking-related cancer mortality that included product interaction terms between ln-transformed Cd and indicator variables for subgroups defined by age (< 55, 55–64, > 64 years), sex (male, female), postmenopausal status (premenopausal, postmenopausal), smoking status (never, ever, current), cigarette pack-years (0, 1–4, 5–19, ≥ 20), and urinary arsenic concentrations (< 7, 7–13, > 13 μg/g) at baseline. We could not conduct interaction analyses for specific cancers because of the relatively small numbers of deaths.

We conducted several sensitivity analyses. First, to account for urine dilution, we used two alternative strategies: adjusting for ln-transformed urinary creatinine concentrations in micrograms per liter instead of dividing by urinary creatinine concentration, and adjusting for the overall mean specific gravity in the study population of 1.019 (McElroy et al. 2007). We restricted the latter analysis to participants without albuminuria or diabetes because specific gravity is inadequate to adjust for dilution if albumin or glucose is present in urine (Chadha et al. 2001; Voinescu et al. 2002). We also estimated associations without accounting for urine dilution. Second, to confirm that the findings were not affected by using age as the time scale, we reevaluated the proportional hazards assumption for Cd after fitting models using calendar time as the time scale and age as a covariate. Third, to account for competing risks by causes of death other than cancer, we estimated proportional hazard regression models according to the method of Fine and Gray (1999), which models the subhazard of the event of interest, reestablishing the direct relationship between the subdistribution of the hazard and the cumulative incidence function. Fourth, to reduce the possibility that prevalent cancers at baseline could affect U-Cd concentrations, we repeated the analyses excluding participants who died of cancer during the first 2 or 5 years of follow-up. Fifth, to evaluate the stability of associations over time, we conducted separate analyses for the first and second decades of follow-up. Finally, because smoking is a major source of Cd and adjusting for smoking might be insufficient to eliminate confounding by smoking, we repeated the analyses excluding current smokers. Findings from all sensitivity analyses were consistent with those reported.

To assess the role of Cd as a possible mediator in the association between tobacco smoke and cancer mortality, we calculated the proportion of additional cases of lung cancer due to tobacco smoking that can be attributed to Cd exposure, using the method proposed by Lange and Hansen (2011), with bootstrap confidence intervals estimated as bias-corrected and accelerated percentile intervals. In brief, we first estimated the direct effect of smoking, as measured by cigarette pack-years, on cancer (direct pathway) using the Aalen additive hazard model. Then, we estimated the indirect effect using two models: *a*) a linear regression with Cd as the dependent variable and number of cigarette pack-years as the independent variable, and *b*) the Aalen additive hazard model for Cd adjusted for cigarette pack-years. We estimated the proportion of lung cancer mortality associated with a 10–cigarette pack-year increase that can be attributed to U-Cd as the ratio of the indirect effect to the total effect.

## Results

During the follow-up period, 2,310 participants died, including 219 women and 155 men whose deaths were attributed to cancer. The most common cause of cancer deaths were lung (*n* = 34) and breast (*n* = 25) cancer in women, and lung (*n* = 43) and prostate (*n* = 16) cancer in men (Table 1). A total of 28 cancer deaths were unspecified (ICD-9 codes: 194–199, 125, and 239). Older participants, those with lower education levels, participants living in North or South Dakota, current smokers, and never drinkers at baseline had higher cancer mortality.

**Table 1:** Baseline characteristics of study participants overall and by cancer mortality status.

Variable	Mortality			*p*-Value^*a*^
	Overall (*n *= 3,792)	Cancer (*n *= 375)	Other causes (*n *= 3,417)	
Age (years)	56.2 ± 0.13	60.2 ± 0.42	55.8 ± 0.14	< 0.001
Men [*n* (%)]	1,538 (40.6)	155 (41.3)	1,383 (40.5)	0.72
Post­menopausal women [*n* (%)]^*b*^	1,733 (76.9)	192 (86.8)	1,541 (75.8)	< 0.001
Arizona [*n* (%)]	1,268 (33.5)	108 (28.8)	1,160 (33.9)	0.05
Oklahoma [*n* (%)]	1,252 (33.0)	121 (32.3)	1,131 (33.1)	0.77
North/South Dakota [*n* (%)]	1,272 (33.5)	146 (38.9)	1,126 (32.9)	0.02
< High school [*n* (%)]	1,799 (47.4)	202 (53.9)	1,597 (46.7)	0.01
Current smoking [*n* (%)]	1,296 (34.1)	161 (42.9)	1,135 (33.2)	< 0.001
Former smoking [*n* (%)]	1,212 (32.0)	113 (30.1)	1,099 (32.2)	0.44
Cigarette pack-years	16.3 ± 0.41	22.7 ± 1.66	15.5 ± 0.41	< 0.001
Never drinking [*n* (%)]	621 (16.4)	75 (20.0)	546 (16.0)	0.01
BMI [kg/m^2^]	30.9 ± 0.10	30.4 ± 0.34	30.9 ± 0.11	0.11
Data are numbers and percentages for categorical variables or mean ± SD for continuous variables. ^***a***^Based on the chi-square test for qualitative variables and analysis of the variance for quantitative variables. ^***b***^Subsample of women (*n *= 2,254).				

The median (IQR) concentration of Cd at baseline was 1.02 (0.60–1.70) μg/L [0.93 (0.61–1.46) μg/g creatinine], with higher levels in participants from North and South Dakota than participants from Arizona or Oklahoma (Table 2). Lower creatinine-corrected U-Cd levels were observed in men, participants < 55 years of age, and participants with a higher level of education. Current smokers and individuals with BMI values < 25 kg/m^2^ showed the highest U-Cd concentrations. U-Cd levels increased with increasing pack-years of smoking in both former smokers [median Cd levels among those smoking ≥ 20 packs/year = 1.36 μg/g creatinine] and current smokers (median Cd concentrations among those smoking ≥ 20 packs/year = 1.57 μg/g creatinine).

**Table 2:** Median (IQR) U-Cd concentrations by participant characteristics at baseline.

Variable	Category	*n*	Median (IQR) (μg/g creatinine)	*p*-Value^*a*^	Median (IQR) (μg/L)	*p*-Value^*a*^
All participants	Total	3,792	0.93 (0.61–1.46)		1.02 (0.60–1.70)
Age (years)	< 55	1,883	0.88 (0.57–1.35)	< 0.001	1.01 (0.58–1.69)	0.26
	55–64	1,166	1.00 (0.65–1.56)		1.06 (0.65–1.73)
	> 64	743	0.98 (0.63–1.53)		0.98 (0.56–1.66)
Sex	Male	1,538	0.71 (0.46–1.08)	< 0.001	0.95 (0.56–1.59)	0.003
	Female	2,254	1.11 (0.74–1.71)		1.06 (0.63–1.78)
Postmenopausal women	Yes	521	1.03 (0.70–1.51)	0.001	1.17 (0.62–1.87)	< 0.001
	No	1,733	1.13 (0.75–1.74)		1.03 (0.63–1.74)
Center	Arizona	1,268	0.82 (0.55–1.22)	< 0.001	0.84 (0.51–1.36)	< 0.001
	Oklahoma	1,252	0.87 (0.57–1.35)		0.96 (0.58–1.62)
	North/South Dakota	1,272	1.13 (0.75–1.80)		1.30 (0.76–2.10)
Education level	< High school	834	1.01 (0.66–1.57)	< 0.001	1.00 (0.60–1.68)	< 0.001
	High school	965	1.01 (0.65–1.59)		1.02 (0.61–1.78)
	> High school	1,993	0.88 (0.57–1.34)		1.02 (0.60–1.67)
Smoking status	Never	1,284	0.88 (0.57–1.36)	< 0.001	0.86 (0.53–1.40)	< 0.001
	Former	1,212	0.79 (0.53–1.22)		0.90 (0.55–1.49)
	Current	1,296	1.14 (0.74–1.73)		1.36 (0.80–2.18)
Cigarette pack-years	0	1,284	0.88 (0.57–1.36)	< 0.001	0.86 (0.53–1.40)	< 0.001
	1–4	931	0.84 (0.54–1.29)		0.92 (0.56–1.56)
	5–19	748	0.93 (0.62–1.44)		1.18 (0.70–1.88)
	≥ 20	829	1.14 (0.76–1.72)		1.33 (0.77–2.19)
Alcohol	Never	621	1.03 (0.67–1.59)	< 0.001	0.96 (0.56–1.66)	0.01
	Former	1,583	0.91 (0.60–1.46)		0.96 (0.58–1.64)
	Current	1,588	0.91 (0.59–1.39)		1.09 (0.64–1.78)
BMI (kg/m^2^)	< 25	591	1.17 (0.75–1.84)	< 0.001	1.19 (0.65–2.05)	< 0.001
	25–30	1,276	0.96 (0.61–1.50)		1.02 (0.61–1.69)
	> 30	1,925	0.86 (0.57–1.30)		0.97 (0.58–1.61)
^***a***^Based on the Kruskall–Wallis exact test.						

After multivariable adjustment (Table 3), the HRs (95% CIs) for overall and for smoking-related cancer mortality comparing the 80th versus the 20th percentile of Cd concentrations in urine were 1.30 (95% CI: 1.09, 1.55) and 1.56 (95% CI: 1.24, 1.96), respectively. The corresponding HRs (95% CIs) for cancers of the lung and pancreas were 2.27 (95% CI: 1.58, 3.27) and 2.40 (95% CI: 1.39, 4.17), respectively. After removing current smokers, the HRs for overall, smoking-related, lung, and pancreatic cancer mortality remained positive but weaker (Table 4). Cd was not significantly associated with other cancers, although the HRs comparing the 80th versus the 20th percentile of Cd concentrations were positive for liver cancer [1.64 (95% CI: 0.81, 3.13)] and lymphohematopoietic tumors [1.40 (95% CI: 0.80, 2.43)].

**Table 3:** HRs (95% CIs) for cancer mortality by U-Cd concentrations (μg/g creatinine).

Outcome	U-Cd concentration			80th vs. 20th percentile^*a*^	*p*_Trend_^*b*^
	≤ 0.70	0.71–1.22	≥ 1.23		
Total cancers (ICD-9 codes 140–208)
Cases/total (*n*/*N*)	77/1,269	142/1,266	156/1,257	375/3,792
Model 1	1 (Referent)	1.80 (1.36, 2.38)	1.94 (1.47, 2.57)	1.36 (1.16, 1.59)	< 0.001
Model 2	1 (Referent)	1.76 (1.32, 2.35)	1.85 (1.36, 2.51)	1.30 (1.09, 1.55)	< 0.001
Smoking-related cancers (ICD-9 codes 140–149, 150–151, 153–155, 157, 161, 162, 180, 188–189, 205)^*c*^
Cases/total (*n*/*N*)	34/1,269	72/1,266	104/1,257	210/3,792
Model 1	1 (Referent)	2.04 (1.36, 3.07)	2.81 (1.90, 4.16)	1.56 (1.28, 1.91)	< 0.001
Model 2	1 (Referent)	2.04 (1.34, 3.11)	2.80 (1.82, 4.31)	1.56 (1.24, 1.96)	< 0.001
Esophagus and stomach cancer (ICD-9 codes 150–151)
Cases/total (*n*/*N*)	11/1,269	6/1,266	7/1,257	24/3,792
Model 1	1 (Referent)	0.55 (0.20, 1.49)	0.68 (0.26, 1.79)	0.63 (0.33, 1.20)	0.16
Model 2	1 (Referent)	0.60 (0.21, 1.68)	0.76 (0.26, 2.23)	0.68 (0.34, 1.38)	0.29
Colon and rectal cancer (ICD-9 codes 153–154)
Cases/total (*n*/*N*)	6/1,269	14/1,266	12/1,257	32/3,792
Model 1	1 (Referent)	2.27 (0.87, 5.93)	1.76 (0.65, 4.75)	1.06 (0.60, 1.86)	0.84
Model 2	1 (Referent)	2.23 (0.82, 6.02)	1.74 (0.60, 5.11)	0.98 (0.51, 1.88)	0.96
Liver and intrahepatic bile ducts (ICD-9 code 155)
Cases/total (*n*/*N*)	4/1,269	7/1,266	10/1,257	21/3,792
Model 1	1 (Referent)	1.79 (0.52, 6.14)	2.83 (0.87, 9.14)	1.51 (0.81, 2.81)	0.20
Model 2	1 (Referent)	2.11 (0.59, 7.55)	3.67 (1.01, 13.32)	1.64 (0.81, 3.13)	0.14
Gall blader and extrahepatic bile ducts (ICD-9 code 156)
Cases/total (*n*/*N*)	3/1,269	5/1,266	3/1,257	11/3,792
Model 1	1 (Referent)	1.56 (0.37, 6.57)	0.94 (0.19, 4.77)	1.13 (0.44, 2.86)	0.80
Model 2	1 (Referent)	1.28 (0.29, 5.67)	0.66 (0.11, 3.90)	0.89 (0.31, 2.54)	0.82
Pancreas (ICD-9 code 157)
Cases/total (*n*/*N*)	12/1,269	—^*d*^	12/1,257	24/3,792
Model 1	1 (Referent)	—	2.00 (0.89, 4.52)	2.00 (1.19, 3.36)	0.009
Model 2	1 (Referent)	—	2.47 (1.01, 6.03)	2.40 (1.39, 4.17)	0.002
Bronchus and lung (ICD-9 code 162)
Cases/total (*n*/*N*)	4/1,269	21/1,266	52/1,257	77/3,792
Model 1	1 (Referent)	4.85 (1.66, 14.1)	10.2 (3.67, 28.4)	2.33 (1.76, 3.09)	< 0.001
Model 2	1 (Referent)	3.39 (1.14, 10.1)	6.65 (2.29, 19.3)	2.27 (1.58, 3.27)	< 0.001
Breast (ICD-9 code 174)
Cases/total (*n*/*N*)	6/504	12/786	7/964	25/2,254
Model 1	1 (Referent)	1.29 (0.48, 3.47)	0.60 (0.20, 1.83)	1.01 (0.51, 1.98)	0.15
Model 2^*e*^	1 (Referent)	1.34 (1.14, 10.1)	0.58 (0.18, 1.83)	1.02 (0.50, 2.07)	0.96
Prostate (ICD-9 code 185)
Cases/total (*n*/*N*)	4/765	8/480	4/293	16/1,538
Model 1	1 (Referent)	1.80 (0.54, 6.00)	0.85 (0.2, 3.48)	0.70 (0.30, 1.62)	0.41
Model 2	1 (Referent)	1.37 (0.40, 4.66)	0.48 (0.11, 2.08)	0.42 (0.16, 1.08)	0.07
Kidney (ICD-9 code 189)
Cases/total (*n*/*N*)	8/1,269	11/1,266	6/1,257	26/3,792
Model 1	1 (Referent)	1.40 (0.56, 3.50)	0.82 (0.28, 2.42)	0.83 (0.44, 1.56)	0.64
Model 2^*f*^	1 (Referent)	1.92 (0.73, 5.01)	1.39 (0.43, 4.58)	1.15 (0.58, 2.31)	0.61
Lymphohematopoietic tissue (ICD-9 codes 200–208)
Cases/total (*n*/*N*)	6/1,269	17/1,266	14/1,257	37/3,792
Model 1	1 (Referent)	2.96 (1.16, 7.52)	2.73 (1.04, 7.20)	1.45 (0.87, 2.40)	0.15
Model 2	1 (Referent)	2.94 (1.12, 7.70)	2.79 (0.99, 7.90)	1.40 (0.80,2.43)	0.24
Model 1, adjusted for sex and age. Model 2, adjusted for sex, age, smoking status (never, former, current), cigarette pack-years (continuous), and BMI (< 25, 25–30, ≥ 30 kg/m^2^). ^***a***^Models comparing the 80th versus the 20th percentiles of U-Cd and associated *p*_trend_ were obtained from Cox proportional hazards models with ln-transformed Cd as a continuous variable, allowing computation of the expected association comparing Cd levels at the the 80th percentile (1.62 μg/g creatinine) to those on the 20th percentile (0.55 μg/g creatinine). ^***b***^*p*_trend_ from the log likelihood ratio test calculated modeling ln-transformed Cd as continuous. ^***c***^Smoking-related cancers: lip, oral cavity, and pharynx (ICD-9 codes 140–149); esophagus (code 150); stomach (code 151); colon and rectum (codes 153–154); liver (code 155); pancreas (code 157); larynx (code 161); trachea, bronchus, and lung (code 162); cervix (code 180); bladder (code 188); kidney (code 189); myeloid leukemia (code 205). ^***d***^Tertiles 1 and 2 were combined into one single group because there was only one case in the first tertile. ^***e***^Model 2 for breast cancer was further adjusted for menopausal status (premenopausal, postmenopausal), parity (0, 1–2, 3–4, ≥ 5), and hormonal replacement therapy (current, past, never use). ^***f***^Model 2 for kidney cancer was further adjusted for estimated glomerular filtration rate (continuous) and hypertension status (yes, no).					

**Table 4:** HRs (95% CIs) for cancer mortality comparing the 80th versus the 20th percentiles of U-Cd concentrations in all participants and in noncurrent smokers (never and former smokers).

Cancer outcome	All participants	Never and former smokers
All cancers
Cases/total (*n*/*N*)	375/3,792	214/2,496
Model 2	1.30 (1.09, 1.55)	1.17 (0.93, 1.48)
Smoking-related
Cases/total (*n*/*N*)	210/3,792	107/2,496
Model 2	1.56 (1.24, 1.96)	1.37 (1.00, 1.87)
Pancreatic
Cases/total (*n*/*N*)	24/3,792	15/2,496
Model 2	2.41 (1.39, 4.17)	2.22 (1.12, 4.40)
Bronchus/lung
Cases/total (*n*/*N*)	77/3,792	17/2,496
Model 2	2.27 (1.58, 3.27)	2.06 (1.15, 3.70)

**Figure 1:**
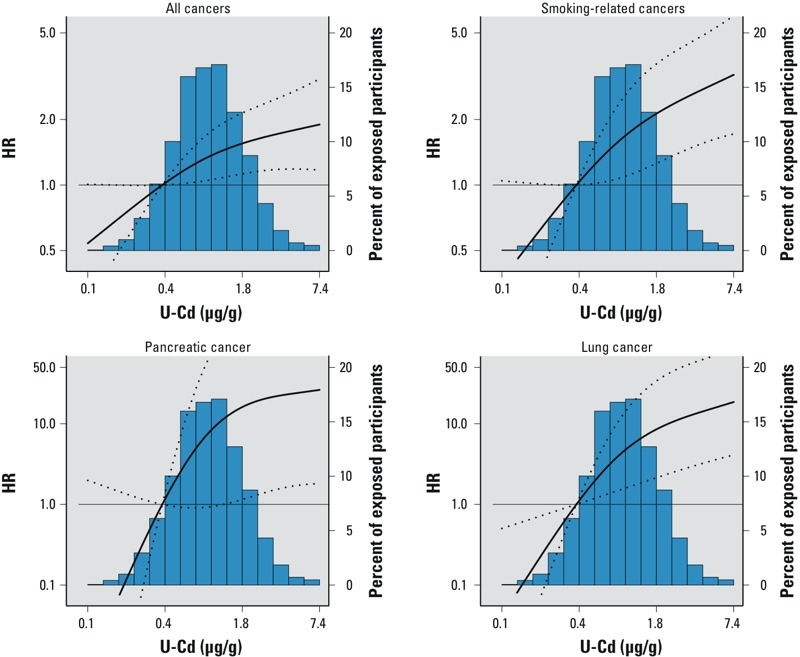
HRs (95% CIs) for overall, smoking-related, pancreatic, and lung cancer mortality based on restricted cubic splines for ln-transformed U-Cd concentrations with knots at the 10th (0.4 μg/g creatinine), 50th (0.93 μg/g creatinine), and 90th (2.15 μg/g creatinine) percentiles. The reference value is set at the 10th percentile of the Cd distribution. HRs were adjusted for sex, age, smoking status, cigarette pack-years, and BMI. Lines represent the HR (thick line) and 95% CIs (dotted lines). The p-value for the linear and nonlinear components of the dose–response relationship were, respectively, 0.03 and 0.26 for all cancers, 0.02 and 0.25 for smoking-related cancers, 0.02 and 0.09 for pancreatic cancer, and 0.01 and 0.10 for lung cancer. The *p*-value for the nonlinear component was estimated using the Wald test.

When modeling the dose–response relationship using restricted cubic splines, we found increased risks with increasing U-Cd concentrations for overall, smoking-related, lung, and pancreatic cancer mortality, with no statistically significant departures from linearity (Figure 1). The associations for overall, smoking-related, lung, and pancreatic cancers were attenuated in models that did not account for urine dilution (see Supplemental Material, Table S1).

**Figure 2:**
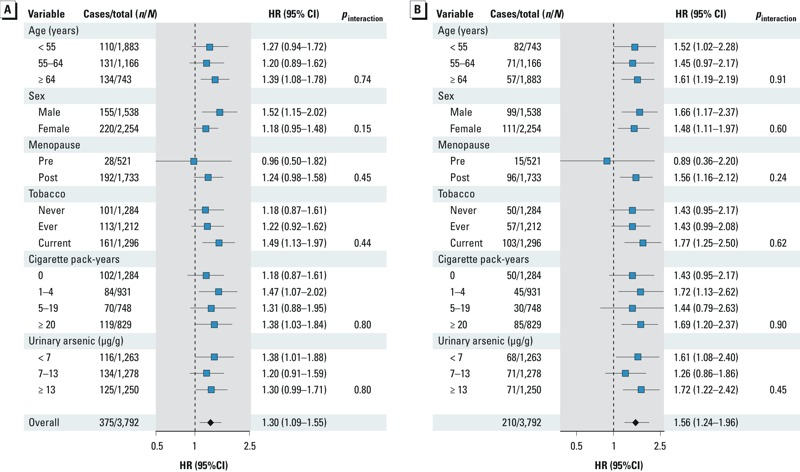
Fully adjusted HRs (95% CIs) for overall (*A*) and smoking-related (*B*) cancer mortality comparing the 80th versus the 20th percentile of Cd (μg/g creatinine) by participant characteristics at baseline.

In subgroup analyses, the fully adjusted HRs for all-cancer mortality and for smoking-related cancer mortality comparing the 80th versus the 20th percentiles of Cd were consistent for all participant subgroups evaluated, including smoking status, although these associations seemed stronger among current smokers (Figure 2).

Analyses investigating Cd as a possible mediator of the association between tobacco smoke and lung cancer mortality suggested that the percentage of cancer deaths due to tobacco smoking that could be attributed to Cd was 9.0% (95% CI: 2.8%, 21.8%), assuming no other mediators in the model.

## Discussion

Low-to-moderate Cd exposure, as measured in urine, was associated with mortality from overall, smoking-related, lung, and pancreatic cancer over almost 20 years of follow-up. The associations remained after adjusting for sociodemographic and behavioral factors, including smoking status and cigarette pack-years at baseline. As expected, the associations for overall, smoking-related, lung, and pancreatic cancer were attenuated when not accounting for urine dilution because urine dilution is an important source of measurement error in our study population, which has a high burden of uncontrolled diabetes (Lee et al. 1995). Our findings are consistent with previous cohort studies showing increased incidence and mortality for overall (Menke et al. 2009; Nawrot et al. 2006), lung (Adams et al. 2012; Nawrot et al. 2006; Verougstraete et al. 2003), and pancreatic cancers (Adams et al. 2012) in association with Cd exposure. Contrary to other studies, however, we found no significant positive association with prostate (Julin et al. 2012b; Kolonel and Winkelstein 1977; Lemen et al. 1976; Sharma-Wagner et al. 2000), breast (Cantor et al. 1995; Gallagher et al. 2010; Julin et al. 2012a), or kidney cancer (Il’yasova and Schwartz 2005), although we had limited power to identify associations because of the small numbers of deaths for these cancers.

Cd exposure induces lung and pancreatic cancer in rodent models (Huff et al. 2007; Waalkes 2003). Proposed mechanisms for Cd carcinogenicity include oxidative stress (Bertin and Averbeck 2006; Hart et al. 1999; Joseph 2009; Patra et al. 2011), inhibition of DNA repair systems (Jin et al. 2003; McMurray and Tainer 2003; Potts et al. 2003), inhibition of apoptosis (Joseph 2009), epigenetic modifications affecting gene transcription (Achanzar et al. 2000; Bertin and Averbeck 2006), and endocrine disruption (Byrne et al. 2009). In human airway epithelial cells, Cd has been shown to promote inflammation through the action of cytokines (Cormet-Boyaka et al. 2012) and increased reactive oxygen species formation (Son et al. 2012). *In vitro*, chronic exposure of human pancreatic duct epithelial cells to Cd resulted in malignant cell transformation with increased secretion of metalloproteinases, increased invasiveness, and increased colony formation (Qu et al. 2012).

Smoking, a cause of several cancers including lung and pancreatic cancer (IARC 2012), is an important source of Cd exposure (Satarug and Moore 2004). In the present study, associations of Cd with lung cancer and pancreatic cancer remained significant after adjusting for smoking status and cigarette pack-years at baseline, suggesting that Cd is an independent risk factor for these tumors, although we cannot discard residual confounding. Moreover, although weaker, the associations remained consistent after excluding participants who were current smokers at baseline. We also hypothesized that Cd could act as a mediator of the association between smoking and lung cancer mortality, and we estimated that Cd exposure via smoking explained 9.0% of the excess lung cancer mortality due to tobacco smoking. Mediation analyses are limited by a series of assumptions, including that there is no unmeasured confounding. Cd is only one of the many carcinogens present in tobacco smoke and we had one single Cd measure, which could be affected by measurement error.

Women have higher Cd internal doses compared with men at similar exposure levels, possibly related to their generally higher gastrointestinal absorption (Vahter et al. 2002). It is unclear, however, if this higher Cd internal dose is associated with worse health outcomes in women compared with men. In the present study there were no significant differences in overall or smoking-related cancer mortality by sex, although associations were somewhat stronger in men. Data from the Swedish Mammography Cohort, a population-based prospective cohort study of 55,987 postmenopausal women followed an average of 12.2 years, recently showed that dietary Cd intake was positively associated with overall breast cancer risk (Julin et al. 2012a). Similarly, results from this same cohort suggested an increased risk of endometrial cancer with increasing Cd intake (Akesson et al. 2008). In the United States, a study based on data from both a case–control sample and from NHANES 1999–2008 found an increased risk of breast cancer in women with U-Cd levels > 0.60 μg/creatinine (Gallagher et al. 2010). In the present study we found no association with breast cancer mortality, similar to what was observed in NHANES III (Adams et al. 2012), although we were limited by the small number of breast cancer deaths (*n* = 25) and by the lack of information on incident cases. We could not evaluate the association between U-Cd and endometrial cancer mortality because only two women in our study population died from this cancer.

Results from the present study do not support an increased risk of prostate cancer mortality with increasing U-Cd concentrations. Rather, we found a nonsignificant inverse association. In occupationally exposed men, some (Lemen et al. 1976; Sharma-Wagner et al. 2000; van der Gulden et al. 1995), although not all (Kazantzis et al. 1988; Pukkala et al. 2009), epidemiological studies have shown a positive association between Cd exposure and prostate cancer incidence and mortality. Inconsistent results have also been reported in nonoccupational studies evaluating the association between U-Cd and prostate cancer incidence (Julin et al. 2012b; Lin et al 2013) or prostate cancer mortality (Adams et al. 2012; Li Q et al. 2011).

Results of a systematic review suggested an increased risk of kidney cancer in Cd-exposed workers (Il’yasova and Schwartz 2005), but evidence from general populations is lacking. Cd has also been proposed as a contributor to liver cancer (Satarug 2012), with supportive evidence from China (Campbell et al. 1990). Finally, there is some animal evidence that Cd could induce tumors of the hematopoietic system (Waalkes and Rehm 1994), although there is no epidemiological evidence to support this relationship. Using data from the Strong Heart Study, we found no association between U-Cd and mortality from kidney cancers, and we observed a positive but nonsignificant association with liver and lymphohematopoietic cancer mortality. The small number of deaths in each type of cancer, however, limited our ability to detect associations.

The present study has other limitations. First, we could not exclude participants with cancer at baseline. Analyses excluding cancer deaths during the first 2 and 5 years of follow-up, however, showed similar results (data not shown). Second, we relied on death certificates to identify the cause of death and had no confirmation from hospital records or a cancer registry. Third, we used a single spot urine sample to measure Cd concentrations. Recent studies have also indicated that U-Cd in populations exposed to low-to-moderate levels of Cd might not reflect chronic Cd exposure (Akerstrom et al. 2013). Finally, we had limited statistical power for individual cancer subtypes and for conducting effect modification analyses.

Strengths of the present study include the prospective design and the long follow-up period, the low rate of losses due to follow-up, and the low LOD for U-Cd (Lee et al. 1990; Scheer et al. 2012). Furthermore, our study provides information on cancer mortality in American Indians, an understudied population whose cancer experience and cancer determinants have not been well described. The high concentrations of U-Cd found in these communities [geometric mean (GM) = 0.70 μg/g creatinine in men, 1.14 μg/g creatinine in women] when compared with the general U.S. adult population during the same time period (GM = 0.28 μg/g creatinine in men, 0.40 μg/g creatinine in women) (Menke et al. 2009) suggest that Cd exposure may be an important environmental risk factor for cancer development among American Indians.

## Conclusions

Our study contributes additional evidence in support of low-to-moderate Cd exposure as a cancer risk factor, including total, lung, and pancreatic cancer. The implementation of population-based preventive measures to decrease Cd exposure, including tobacco control measures (Tellez-Plaza et al. 2012), reduction of dust in homes (Hogervorst et al. 2007), and decrease of the transfer of Cd from soil to plants used for human consumption by, for example, maintaining agricultural soil pH close to neutral (Nawrot et al. 2010), could contribute to reducing the burden of cancer.

## Supplemental Material

(102 KB) PDF
