# Supplementary material for: Cadmium Exposure and Cancer Mortality in a Prospective Cohort: The Strong Heart Study
Source: Environ Health Perspect. 2014 Feb 14;122(4):363–70. doi: 10.1289/ehp.1306587 (PMC3984227; doi:10.1289/ehp.1306587)
Supplement: (102 KB) PDF [file ehp.1306587.s001.pdf]

Environmental Health Perspectives doi:10.1289/ehp.1306587

Supplemental Material
Cadmium Exposure and Cancer Mortality in a Prospective Cohort: The Strong Heart Study

Esther García-Esquinas, Marina Pollan, Maria Tellez-Plaza, Kevin A. Francesconi, Walter Goessler,

Eliseo Guallar, Jason G. Umans, Jeunliang Yeh, Lyle G. Best, and Ana Navas-Acien.

Supplemental Material, Table S1. Hazard ratios (95%CI) for cancer mortality comparing the 80th vs. 20th percentile of U-Cd levels (µg/L) without accounting for urine dilution (model A), adjusting for urine creatinine as a separate variable (model B) and dividing urine cadmium by urine creatinine (model C).

**Cancer type**	**Cases/Total**	**Model A**	**Model B**	**Model C**[a]
Total cancer	375/3,792	1.11 (0.93, 1.33)	1.37 (1.10, 1.70)	1.30 (1.09, 1.55)
Smoking related cancers	210/3,792	1.25 (0.99, 1.59)	1.78 (1.35, 2.35)	1.56 (1.24, 1.96)
Esophagus/Stomach cancer	24/3,792	0.95 (0.46, 1.94)	0.68 (0.28, 1.68)	0.68 (0.34, 1.38)
Colon/Rectal cancer	32/3,792	0.86 (0.46, 1.60)	0.84 (0.38, 1.88)	0.98 (0.51, 1.88)
Liver/Intrahepatic bile ducts	21/3,792	0.93 (0.44, 1.96)	1.50 (0.64, 3.51)	1.64 (0.81, 3.13)
Gallbladder/Extrahepatic bile ducts	11/3,792	1.07 (0.36, 3.16)	1.00 (0.26, 3.88)	0.89 (0.31, 2.54)
Pancreas	24/3,792	1.34 (0.66, 2.74)	2.62 (1.26, 5.44)	2.40 (1.39, 4.17)
Bronchus/Lung	77/3,792	1.62 (1.07, 2.46)	3.27 (2.13, 5.03)	2.27 (1.58, 3.27)
Breast	25/2,254	1.02 (0.52, 1.99)	0.98 (0.42, 2.30)	1.02 (0.50, 2.07)
Prostate	16/1,538	1.01 (0.38, 2.69)	1.18 (0.31, 4.47)	0.42 (0.16, 1.08)
Kidney	26/3,792	1.24 (0.62, 2.48)	1.21 (0.52, 2.82)	1.15 (0.58, 2.31)
Lymphohematopoietic tissue	37/3,792	0.83 (0.46, 1.47)	0.97 (0.47, 2.04)	1.40 (0.80, 2.43)

[a] Model C corresponds to Model 2 in Table 3.

All models are adjusted for sex, age, smoking status , pack-years and BMI, and account for age by using age as the time scale and age at baseline examination treated as staggered entries.
